# New Technologies in Endourology and Laser Lithotripsy: The Need for Evidence in Comprehensive Clinical Settings

**DOI:** 10.3390/jcm12175709

**Published:** 2023-09-01

**Authors:** Luigi Candela, Etienne X. Keller, Amelia Pietropaolo, Francesco Esperto, Patrick Juliebø-Jones, Esteban Emiliani, Vincent De Coninck, Thomas Tailly, Michele Talso, Senol Tonyali, Emre T. Sener, B. M. Zeeshan Hameed, Lazaros Tzelves, Ioannis Mykoniatis, Arman Tsaturyan, Andrea Salonia, Eugenio Ventimiglia

**Affiliations:** 1 Division of Experimental Oncology, Unit of Urology, URI, IRCCS Ospedale San Raffaele, Via Olgettina 60, 20132 Milan, Italy; 2 School of Medicine, Vita-Salute San Raffaele University, 20132 Milan, Italy; 3 EAU Young Academic Urologists (YAU) Urolithiasis and Endourology Working Group Arnhem, NL-6803 Arnhem, The Netherlands; 4 Department of Urology, University Hospital Zurich, University of Zurich, 8006 Zurich, Switzerland; 5 Department of Urology, University Hospital Southampton NHS Foundation Trust, Southampton SO16 6YD, UK; 6 Department of Urology, Campus Biomedico University of Rome, 00128 Rome, Italy; 7 Department of Urology, Haukeland University Hospital, N-5021 Bergen, Norway; 8 Department of Urology, Fundacio Puigvert, Autonomous University of Barcelona, 08193 Barcelona, Spain; 9 Department of Urology, AZ Klina, 2930 Brasschaat, Belgium; 10 Department of Urology, University Hospital Ghent, 9000 Gent, Oost-Vlaanderen, Belgium; 11 ASST Fatebenefratelli Sacco, Department of Urology, Luigi Sacco University Hospital, Via Giovanni Battista Grassi, 74, 20157 Milano, Italy; 12 Urology, Istanbul University School of Medicine, Topkapı, Turgut Özal Millet Cd, Istanbul 34093, Turkey; 13 Department of Urology, Marmara University School of Medicine, Istanbul 34854, Turkey; 14 Department of Urology, Father Muller Medical College, Karnataka 575002, India; 15 Institute of Urology, University College Hospital London, London NW1 2BU, UK; 16 Department of Urology, Aristotle University of Thessaloniki, 541 24 Thessaloniki, Greece; 17 Department of Urology, Erebouni Medical Center, 0087 Yerevan, Armenia

Flexible ureteroscopy (fURS) with laser lithotripsy is currently the gold standard surgical treatment for ureteral and kidney stones with a maximum diameter of 2 cm [1]. For nearly 25 years, low-power (LP) Holmium:YAG (Ho:YAG) laser has been the technology of reference in this field to achieve satisfactory outcomes in terms of safety and efficacy for all types of stones [2]. While LP Ho:YAG laser generators have demonstrated acceptable intraoperative and postoperative complication rates and stone-free rates (SFR), this technology still has certain limitations, especially regarding lithotripsy efficiency (measured in J/mm^3^) and lithotripsy speed (mm^3^/s). The need to overcome the limitations of LP Ho:YAG laser lithotripsy has prompted major technological advancements in the field of laser technology for endoscopic lithotripsy. The introduction of high-power (HP) Ho:YAG laser generators up to 150 W, the implementation of pulse modulation for the Ho:YAG laser beam, and the marketing of both thulium fiber laser (TFL) and pulsated Tm:YAG laser generators have dramatically increased the number of available options. However, these novelties have not always been supported by an adequate level of scientific evidence, especially in real-life clinical scenarios. Several pre-clinical studies have shown the potential advantages of these new laser technologies for endoscopic lithotripsy, but there is an absence of consistent in vivo evidence.

As opposed to LP Ho:YAG laser generators, HP variants may achieve high frequencies, reaching up to 120 Hz, thus leading to potentially faster stone ablation. This has important implications in terms of the choice of parameters, lithotripsy technique, and perioperative outcomes [3]. A recent systematic review reported that HP Ho:YAG laser lithotripsy had a shorter mean operative time compared to LP; however, no significant differences were found in terms of surgical complications and SFR. Do we have enough evidence to draw conclusions on this matter? Considering the aforementioned report, only one non-randomized comparative study was available, and the remaining studies were single-arm case series; therefore, there was a relevant risk of bias in the pooled estimate [4]. In a randomized controlled trial comparing LP and HP Ho:YAG laser settings during ureteroscopic laser lithotripsy, Shrestha and colleagues found that HP lithotripsy did not significantly increase lithotripsy speed, whereas it delivered more energy compared to LP lithotripsy for treating an equal amount of stone volume (higher J/mm^3^) [5].

In addition to the implementation of HP lithotripsy, does pulse modulation play a significant role as well? Despite the possibility of configuring the laser for both short and long pulse modalities for stone fragmentation and dusting lithotripsy technique [6], the Moses Technology^TM^ (MT, Boston Scientific, Marlborough, MA, USA) made its entrance into the endourological armamentarium in 2017. Several other similar options, such as Virtual Basket^TM^ (Quanta System, Samarate, Italy), Advanced Mode^TM^ (Dornier MedTech, Munich, Germany), and Stabilization Mode^TM^ (Olympus, Shinjuku, Tokyo, Japan), were later developed. The underlying idea is to split a single laser pulse into two sub-pulses with different peak power. The first sub-pulse generates a vapor bubble in the fluid medium, enabling a second sub-pulse to reach the target without dissipating its energy in the medium, thereby maximizing the effect on the target and reducing stone retropulsion [7]. However, despite the appealing concept and promising pre-clinical laboratory results, this technology is not yet solidly supported by high-level clinical evidence [8]. Moreover, MT effects in terms of lithotripsy efficiency share similar characteristics with low-peak power Ho:YAG laser pulses [9]. Similar conclusions were reported in a recent systematic review conducted by Corrales et al., including studies on the more recently launched MT 2.0 [10]. There is still conflicting evidence regarding pulse-modulated Ho:YAG lithotripsy: a recent retrospective comparative study conducted in a high-volume proficient endourological centre found that the use of HP Ho:YAG laser with MT was significantly faster for stone lithotripsy and reduced both operative time and the need for a second procedure compared to LP Ho:YAG laser [11].

The latest innovation in the field of endoscopic laser lithotripsy is represented by TFL, which is rapidly spreading worldwide as an excellent alternative to the Ho:YAG laser. This laser technology largely differs from Ho:YAG laser in several physical and technical aspects [12]. Briefly, with TFL, it is possible to obtain the following: (i) a wider possibility of laser setting with potential very low energies (0.025 J) and high frequencies (2000 Hz); (ii) the use of thinner laser fibers and thus better endoscope deflection and irrigation, (iii) less stone retropulsion; (iv) higher ablation speed for any stone composition, which is two to five times faster than Ho:YAG even with MT; (v) thinner dust particles; and (vi) higher postoperative stone-free rate [12,13]. However, most of the evidence regarding the advantages of TFL over Ho:YAG stems from pre-clinical studies, and the clinical benefits in real-life scenarios are still debated and controversial [13,14,15,16].

Overall, despite the improvement in available technologies for endoscopic laser lithotripsy and the broader field of endourology [17], there is a need for robust clinical data that will eventually support clinical decision making.

Furthermore, when treating stone disease, it is important to remember that there is much more that we can do beyond properly using the available technology. For instance, adequate knowledge of the biology of lithogenesis and the possible measures aimed at improving the management of patients with stone disease significantly improves the clinical outcomes of patients [18].

In conclusion, a surge of technological innovations and improvements has recently occurred in the field of endoscopic laser lithotripsy. However, clinical evidence regarding the benefits in terms of surgical outcomes in real-life scenarios is still lacking, as most of the available results originate from pre-clinical studies. Beyond the importance of technology, we emphasize the importance of patient metabolic assessment in the pursuit of obtaining and maintaining a stone-free status.
